# Identification of multiple prognostic biomarker sets for risk stratification in SKCM

**DOI:** 10.3389/fbinf.2025.1624329

**Published:** 2026-01-07

**Authors:** Shivani Malik, Ritu Tomer, Akanksha Arora, Gajendra P. S. Raghava

**Affiliations:** Department of Computational Biology, Indraprastha Institute of Information Technology, New Delhi, India

**Keywords:** skin cutaneous melanoma, overall survival, survival analysis, machine learning, prognostic biomarker

## Abstract

**Introduction:**

The majority of available transcriptomics-related cancer prognosis studies strive to define one collection of biomarkers that can be used to predict high-risk patients. However, using a single biomarker profile could restrict its strength and applicability to diverse groups of patients. In order to fill this gap, we discuss the prospect of determining several, discrete sets of prognostic biomarkers in Skin Cutaneous Melanoma (SKCM). Our search identifies various genes including CREG1, PCGF5 and VPS13C whose expression pattern depicts significant correlations with overall survival (OS) in SKCM patients.

**Methods:**

We developed machine learning-based prognostic models using SKCM gene expression data to predict 1-, 3-, and 5-year overall survival. Advanced feature selection approaches were applied to identify prognostic biomarkers. The primary biomarker set consisted of 20 genes selected using state-of-the-art feature selection techniques. Machine learning classifiers were trained to distinguish high-risk from low-risk patients using these biomarkers. The process was systematically repeated to identify seven independent biomarker sets, each containing 20 unique genes without overlap. Model performance was evaluated using AUC and Cohen's Kappa metrics on an independent test dataset. Validation was further performed using the GEO dataset GSE65904, employing subsets of biomarkers from the primary and third sets.

**Results:**

The primary biomarker-based prognostic model demonstrated strong predictive ability, achieving an AUC of 0.90 and a Kappa of 0.58 in identifying high-risk SKCM patients. A second independent 20-gene set, with no overlap with the first, produced an AUC of 0.89 and Kappa of 0.56. Across all seven biomarker sets, performance ranged from 0.84 to 0.91 (AUC) and 0.48 to 0.64 (Kappa). Notably, the fifth biomarker set yielded the highest performance with an AUC of 0.91 and Kappa of 0.64. External validation confirmed the predictive utility of selected biomarkers where genes from the primary set achieved an AUC of 0.83 on GSE65904. While genes from the third set achieved an AUC of 0.86 on the same dataset.

**Discussion:**

Our results show that only one gene-expression signature is not sufficient to predict SKCM prognosis. Alternatively, high-risk patients can be accurately predicted using multiple independent biomarker sets providing flexibility in both clinical and computational practices. The high similarity in the results of all seven sets (AUC 0.84-0.91; Kappa 0.48-0.64) signifies the stability and strength of the method. The external validation of these biomarkers with GEO data also helps to confirm the reliability of these biomarkers and hints at their potential wider applicability. This work facilitates transparency by ensuring that all the data and code is publicly accessible (https://github.com/raghavagps/skcm_prognostic_biomarker), which also promotes future developments in creating multi-signature prognostic tools in melanoma.

## Introduction

Skin Cutaneous Melanoma accounts for around 3%–5% of all malignancies and is a very aggressive tumor that arises from melanocyte cells and primarily affects the skin. However, it can also occur in mucous membranes and internal organs (Wang et al., 2022; Sinagra and Sciume, 2020). The third most frequent type of skin cancer, skin cutaneous melanoma, affects 6.8%–20% of cases (Wang and Liu, 2021). According to projections, there will be 2,001,140 new cases of cancer and 611,720 cancer-related fatalities in the US in 2024. Additionally, there will likely be over 8,000 deaths and 100,640 new cases of SKCM (Siegel et al., 2024). Globally, the incidence of melanoma continues to rise, driven primarily by environmental factors, particularly excessive ultraviolet (UV) radiation exposure, which plays a central role in melanoma carcinogenesis (Tracey and Vij, 2019). Human skin color is primarily determined by the amount of melanin, with darker skin containing larger melanocytes that produce more melanin, offering protection against UV radiation (Kaidbey et al., 1979; Aoude et al., 2014).

**FIGURE 1:**
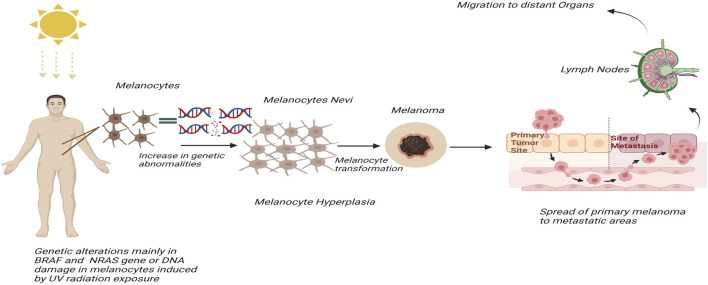
Diagram illustrating the progression of melanoma. UV radiation exposure leads to genetic alterations in melanocytes, causing an increase in genetic abnormalities. Melanocytes transform into nevi and then into melanoma. This melanoma spreads from the primary tumor site to distant organs via lymph nodes, showing the metastasis process.

SKCM can originate from benign melanocytic nevi, including dysplastic, acquired, or congenital forms, or may develop independently. In recent years, both its prevalence and mortality rates have risen significantly. Surgical excision remains the most effective treatment, highlighting the importance of early diagnosis and intervention (Merighi et al., 2009; Kardynal and Olszewska, 2014; Christodoulou et al., 2021; Seitz et al., 2021). Advances in genomic analysis and high-throughput technologies have provided deeper insights into melanoma biology, improving detection and treatment strategies (Scatena et al., 2021). The malignant transformation of melanocytes is driven by genetic alterations, particularly in the BRAF and NRAS genes (Guan et al., 2015) shown in Figure 1. These mutations are central to melanoma development and progression, influencing tumor behavior and patient response to therapies (Garman et al., 2017). Despite significant progress in uncovering the genetic underpinnings of melanoma, late-stage diagnosis remains a major obstacle, limiting the effectiveness of treatments such as surgery, immunotherapy, and targeted therapies (He and Wang, 2023).

Recent studies have focused on identifying invasion-related biomarkers for individualized treatment and prognosis prediction in SKCM. One such study analyzed 124 invasion-associated genes (IAGS) and selected 20 prognostic genes to develop a gene expression-based model (Yang et al., 2023). Similarly, another study used transcriptome data from TCGA to find a predictive signature based on m6A-related lncRNAs (Lin et al., 2024). Another study developed a 10 ferroptosis-related gene (FRG) signature for predicting overall survival in SKCM (Ping et al., 2022). Additionally, eight immune-related lncRNA signatures were identified as a prognostic model for melanoma and validated using Kaplan-Meier analysis (Xiao et al., 2021). In recent studies, a prognostic signature comprising seven NLR-related genes was identified using LASSO-Cox analysis. This signature effectively stratified SKCM patients into high and low-risk groups, demonstrating a strong correlation with overall survival outcomes. Additionally, it revealed distinct immune microenvironment patterns characterized by the activation of inflammatory and interferon pathways and showed potential for predicting responses to immune checkpoint blockade therapy (Geng et al., 2023). A chemokine-related 14-gene prognostic model was developed, revealing distinct immune characteristics and stratification between low- and high-risk groups. This model demonstrates potential in predicting immunotherapy outcomes and chemotherapy efficacy and aiding clinical decision-making for SKCM patients (Ding et al., 2023). While these studies provide valuable insights, they typically focus on a limited set of biomarkers within a specific context, as shown in the Supplementary Table S1.

**FIGURE 2:**
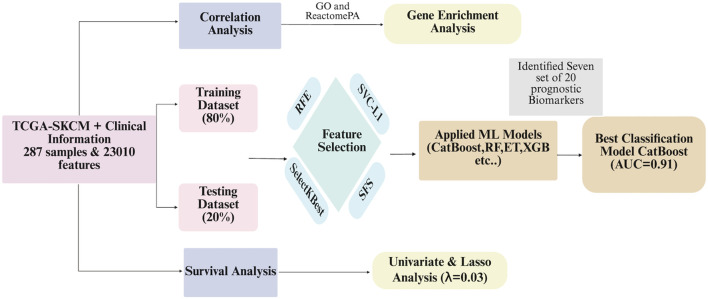
Flowchart depicting an analytical process using TCGA-SKCM clinical information with 287 samples and 23,010 features. It divides data into training (80%) and testing (20%) datasets, undergoes correlation and gene enrichment analysis. Feature selection uses methods like RFE, SVC-L1, SelectKBest, and SFS. Machine learning models applied include CatBoost, RF, ET, and XGB. CatBoost model achieves the best classification with an AUC of 0.91. Survival analysis, univariate and Lasso analysis follow with a lambda of 0.03. Seven sets of twenty prognostic biomarkers are identified.

Previous studies have mostly focused on pathway-related biomarkers. To the best of our knowledge, these methods typically identify only a single set of biomarkers. Moreover, they usually select prognostic biomarkers from a limited subset of data, such as genes related to invasion, immune response, or lncRNAs. In this study, we take a novel approach by attempting to generate biomarker sets from all available genes rather than focusing on just a subset. Additionally, we select a secondary set from the remaining genes after identifying an initial set of biomarkers. This process is repeated, leading to the identification of seven distinct sets of prognostic biomarkers. The pipeline of the study is shown in Figure 2.

## Methodology

### Data collection

Transcriptomic profiling and clinical data were retrieved from The Cancer Genome Atlas (TCGA), a major cancer research database (Yan et al., 2022) using the TCGAbiolinks package from BiocManager in R. The retrieved expression data, which included 473 samples and 60,660 genes, was normalized to Transcripts Per Kilobase Million (TPM) values before downstream analysis using R script. The new dataset GSE65904, comprising 214 samples, was used to confirm the prognostic value of the selected biomarker sets.

### Preprocessing of data

We first removed duplicate features and filtered out genes with more than 50% zero expression values. Using the caret package in R, low-variance features were removed to retain only informative genes, leaving 23,009 genes for further analysis. The gene expression data were then merged with clinical data to identify a cohort of 287 patients with available survival information. For the GEO dataset with available Disease-Specific Survival (DSS) times, 210 samples were selected. Among the seven predefined biomarker sets, 15 genes overlapped with the primary set, 10 with the second set, and 12 with the third set. For each overlapping subset, predictive models were trained and validated using the same cross-validation and hyperparameter tuning strategy.

### Z-score scaling

In order to ensure that each feature has a mean of 0 and a standard deviation of 1, this transformation standardizes the data by subtracting the mean of each feature and dividing it by its standard deviation. By standardizing the data size, this technique makes it possible to compare features more accurately (Tihagam and Bhatnagar, 2023).

### Statistical methods

#### Correlation analysis

The associations between gene expression and OS time were evaluated using Pearson correlation analysis in Base R. False Discovery Rate (FDR) correction was applied using the Benjamini–Hochberg method to account for multiple hypothesis testing. Genes with a p-value and FDR <0.05 were considered significant, providing insights into their potential impact on SKCM patient survival (Ma and Xie, 2024). Positively correlated genes were further analyzed using Gene Ontology (GO) (Wang and Yang, 2022) and Reactome pathway analysis (Fabregat et al., 2017) to identify enriched biological processes and pathways relevant to melanoma progression, utilizing the gseapy package in Python.

### Survival analysis

Patients were divided into high- and low-risk groups based on the median overall survival (OS) time. Univariate Cox regression analysis was conducted using the Cox proportional hazards model (Yang R.-H. et al., 2021). Significant genes were selected based on p-values <0.05 using the survival package in R. Subsequently, LASSO regression analysis (Huang et al., 2022) was performed using the glmnet package in R to identify prognostic genes, with an optimal lambda value of 0.03. Depending on the formula:

$$Risk\;score=\sum_{1}^{n}genei*expression\left(genei\right)$$

Identified prognostic genes were then analyzed using the Drug Gene Interaction Database (DGIdb) (Cannon et al., 2024) to predict potential therapeutic agents based on known drug-gene interactions.

### AI-based methods

We employed various feature selection techniques to identify relevant genes for the prognostic model. Overall survival time was categorized into four classes: Class 0 (0–1 year), Class 1 (one to three years), Class 2 (three to five years), and Class 3 (>5 years).

### Feature selection techniques

To identify the most relevant features for the prognostic model, we applied several established feature selection methods, including Linear Support Vector Classification with L1 penalty (SVC-L1), Recursive Feature Elimination (RFE), Sequential Feature Selection (SFS), and SelectKBest. These methods have been successfully employed in similar studies (Ma and Huang, 2008; Kamkar et al., 2016; Jiang et al., 2023).

### Identification of the primary set of biomarkers

We utilized a variety of feature selection techniques, starting with the Recursive Feature Elimination algorithm (Darst et al., 2018), which recursively ranks features based on their relevance and selects the optimal subset. In the second method, SelectKBest, we applied the f_classif evaluation function to identify the K most relevant features. We also used Linear Support Vector Classification with L1 regularization, which selects features by penalizing less important ones, achieving sparsity, and improving model performance (Bhalla et al., 2019). Specifically, we obtained the absolute values of SVC-L1 coefficients and used the mean coefficient value as a threshold to select features with importance scores above the mean. Sequential Feature Selection was employed to add or remove features to optimize performance sequentially. These techniques helped identify a robust initial biomarker set for further analysis using the Scikit-learn package (Pedregosa et al., 2011).

### Identification of additional biomarker sets

**FIGURE 3:**
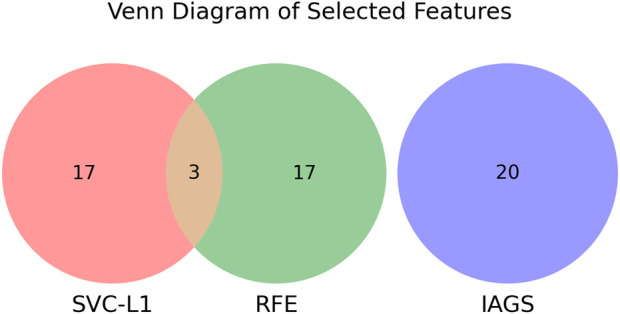
Venn diagram showing selected features from SVC-L1, RFE, and IAGS. SVC-L1 has 17 unique features, RFE has 17 unique features, and IAGS has 20 unique features. Three features overlap between SVC-L1 and RFE.

**FIGURE 4:**
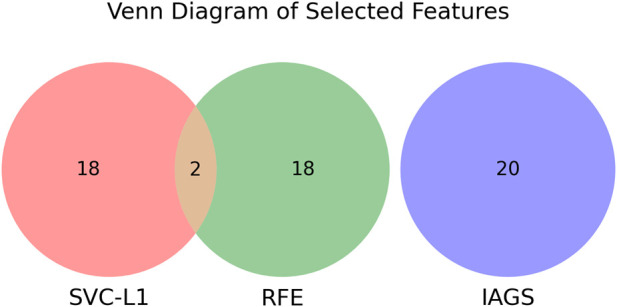
Venn diagram illustrating the overlap of selected features among three methods: SVC-L1, RFE, and IAGS. SVC-L1 contains 18 unique features, RFE contains 18 unique features, and IAGS has 20 unique features. The overlap between SVC-L1 and RFE has 2 features in common.

For the secondary biomarker set, features identified in the primary set were excluded before applying the SVC-L1 method to ensure the independence of the sets. In the case of the third biomarker set, invasion-associated genes, along with features previously identified through RFE and SVC-L1 in earlier analyses, were systematically removed prior to the selection process. This process is visualized in Figures 3, 4, where the Venn diagram highlights the common and distinct features across the biomarker sets and invasion-associated genes. This approach facilitated the identification of non-overlapping and distinct biomarker sets for further analysis.

### ML models

Class imbalance was addressed using the Synthetic Minority Over-sampling Technique (SMOTE), which was applied due to the severity of class imbalance in the dataset. The original class distribution was: Class 0 (0–1 year): 29 patients, Class 1 (one to three years): 110 patients, Class 2 (three to five years): 50 patients, and Class 3 (>5 years): 98 patients. After applying SMOTE, each class was balanced to 110 patients, resulting in a total of 440 samples.

To evaluate model robustness and prevent overfitting, stratified five-fold cross-validation was performed with an 80:20 train-test split, where one sample from every five-sample block was held out as an independent test set, and the remaining samples used for training; this process was repeated to ensure every sample served as a test case. This split yielded 352 samples in the training set and 88 samples in the test set, with each class represented by approximately 22 patients in the test set.

Multiple machine learning algorithms including Multi-Layer Perceptron (MLP), Decision Tree (DT), Extreme Gradient Boosting (XGB), Support Vector Machine (SVM), Extra Trees Classifier (ET), Random Forest (RF), k-Nearest Neighbors (KNN), Light Gradient Boosting Machine (LightGBM), Categorical Boosting (CatBoost), Adaptive Boosting (AdaBoost), and Logistic Regression (LR) were trained and compared on different feature sets to identify robust prognostic biomarkers. Hyperparameters for each model were optimized using grid search within a 5-fold cross-validation on the training set to prevent overfitting. Among the models tested, CatBoost achieved the best performance. The hyperparameters for CatBoost were set as (iterations = 800, depth = 12, learning_rate = 0.2, random_state = 42, cat_features = [], and verbose = 1). This model was then applied to the selected feature sets, and performance was evaluated on the independent test set using accuracy, AUC, and other relevant metrics.

### Ensemble models

We used a voting classifier by utilizing multiple base classifiers, i.e., RF, ET, and LightGBM, to make individual predictions. We used hard voting, where the majority of the votes from the classifiers determined the final class prediction. This method helps aggregate the strengths of different models to achieve better generalization. We also applied a stacking classifier and trained several different base models, including ET and RF. The predictions of these base models were used as inputs to LR as a meta-model, which was trained to combine the base model outputs and make the final prediction. We tried catBoost as a meta-model and RF, ET, and XGB as base models. This two-level architecture leverages the strengths of multiple models by allowing the meta-model to learn how to best combine their predictions.

## Results

### Correlation analysis

Our univariate Cox regression analysis identified 4,324 genes significantly associated with overall survival time. After applying FDR correction using the Benjamini–Hochberg method, 2,667 genes remained statistically significant confirming that our findings are robust. We selected the top genes with the most significant positive and negative correlations, as shown in Table 1. Specifically, the genes CREG1 and PCGF5 exhibited positive correlations with OS with correlation coefficients of 0.40 and 0.38, respectively. Conversely, the genes AC008687.4 and TTYH2 showed negative correlations with OS, with coefficients of −0.23 and −0.23, respectively. A positive correlation suggests that higher expression levels of these genes are associated with longer survival, whereas a negative correlation indicates that higher expression levels are linked to poorer survival outcomes. The Pearson correlation coefficient (r) was used to quantify these associations.

**TABLE 1:** List of top genes with strongest positive and negative correlation with overall survival time, including correlation coefficients, p-values and FDR value.

Gene	Coef	p-value	FDR	Type of correlation
CREG1	0.40	3.63E-12	8.35E-08	Positive
PCGF5	0.38	4.47E-11	5.14E-07	
VPS13C	0.36	2.06E-10	1.58E-06	
CPD	0.35	1.17E-09	6.73E-06	
BBX	0.35	1.64E-09	7.56E-06	
LRRK2	0.34	2.36E-09	8.02E-06	
AC008687.4	−0.23	8.28E-05	0.00348	Negative
TTYH2	−0.23	0.00010	0.00403	
G6PC3	−0.21	0.00025	0.00683	
BOK	−0.21	0.00033	0.00818	
GSTP1	−0.21	0.00043	0.00958	
NUDT8	−0.20	0.00049	0.01037	

**FIGURE 5:**
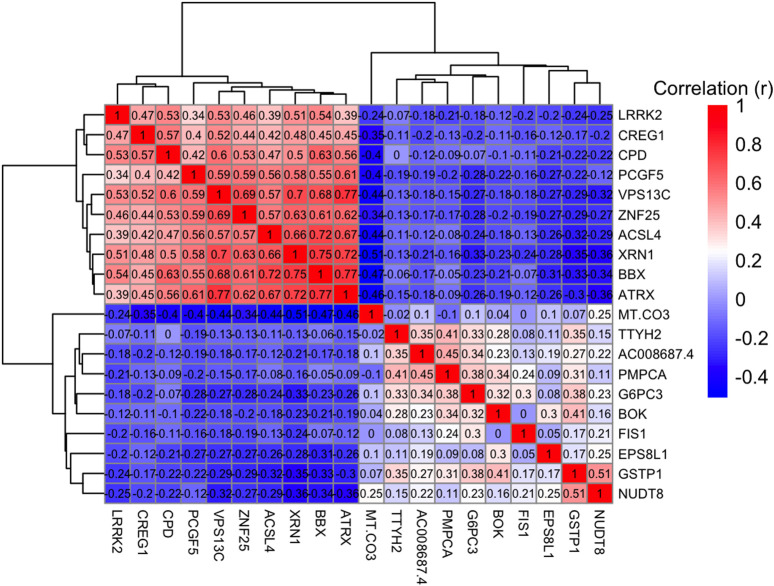
Heatmap displaying correlation values between various gene expressions, ranging from −0.4 to 1. Red indicates positive correlation; blue indicates negative correlation. Dendrograms on top and left organize the genes based on similarity.

**FIGURE 6:**
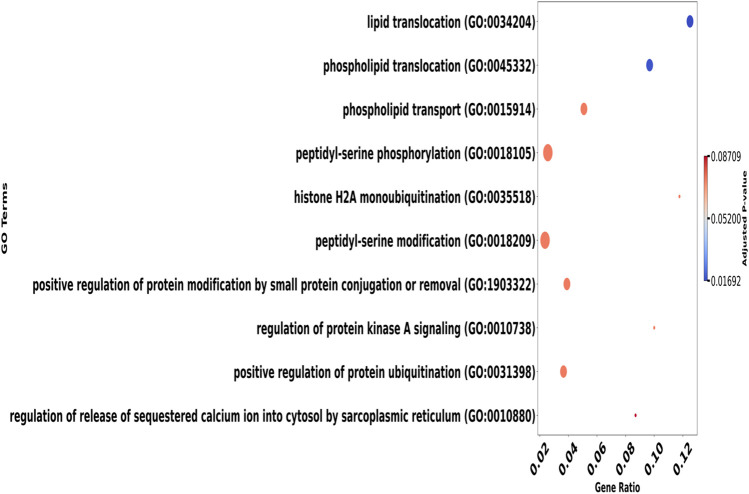
Dot plot illustrating gene ontology (GO) terms against the gene ratio, with adjusted p-values indicated by color. Larger dots represent terms with higher gene ratios. The gradient legend on the right ranges from blue to red, signifying p-values from 0.01692 to 0.08709.

**FIGURE 7:**
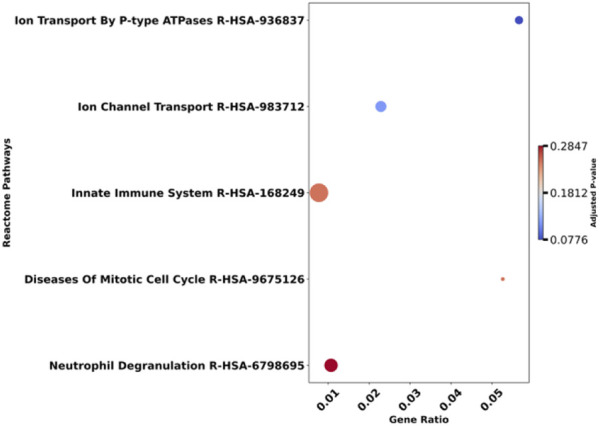
Dot plot illustrating gene ontology (GO) terms against the gene ratio, with adjusted p-values indicated by color. Larger dots represent terms with higher gene ratios. The gradient legend on the right ranges from blue to red, signifying p-values from 0.0776 to 0.2847.

We selected the top genes positively and negatively correlated with overall survival and visualized the relationships among these genes using a heatmap. The heatmap displays pairwise Pearson correlation coefficients between gene expression levels across all samples. Strong positive correlations (red) indicate genes with similar expression patterns, whereas strong negative correlations (blue) indicate genes with opposite expression patterns. Numbers in each cell represent the correlation coefficient (r). This visualization allows identification of clusters of co-expressed genes that may be involved in related biological pathways influencing patient prognosis shown in Figure 5. Afterward, we selected 50 positively correlated genes with high expression levels for enrichment analysis. Significant Gene Ontology (GO) terms included 590 biological processes (BP), 79 cellular components (CC), and no molecular functions (MF). Notable BP enrichments included 'lipid translocation’ (p = 0.00002) and 'phospholipid translocation’ (p = 0.00006), while CC analysis highlighted 'mitochondrial outer membrane’ (p = 0.0002) and 'organelle outer membrane’ (p = 0.0004) are shown in Figure 6. Molecular function analysis revealed significant enrichment in ‘cysteine-type endopeptidase inhibitor activity’ (p = 0.001) and ‘protein serine/threonine kinase activity’ (p = 0.001). Additionally, 246 Reactome pathways were identified, with significant enrichment in pathways such as 'Ion Transport By P-type ATPases’ (p = 0.0003) and 'Ion Channel Transport’ (p = 0.0009). The top 5 GO (BP) terms and Reactome pathways are shown in Figure 7.

### Survival analysis

**FIGURE 8:**
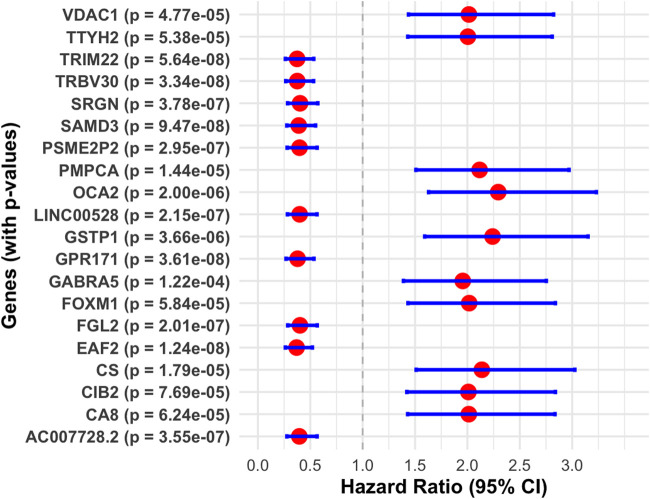
Forest plot displaying hazard ratios and 95% confidence intervals for various genes, each labeled with p-values. Red dots represent hazard ratios, with blue lines indicating confidence intervals. The x-axis presents hazard ratios ranging from 0.0 to 3.0.

Univariate Cox regression analysis was performed to evaluate the association between gene expression and overall survival. Patients were divided into two groups based on the median OS time, categorizing them into low-risk (G1) and high-risk (G2) groups for each gene. A total of 4,324 genes were identified as statistically significant (p-value <0.01). Among these, 1,264 genes had a hazard ratio (HR) greater than 1, indicating a potential risk, while 3,060 genes had an HR less than 1, suggesting a protective effect. The top genes with HR values are highlighted in Figure 8, while a detailed summary of these findings is presented in Table 2.

**TABLE 2:** Hazard ratio (HR)-based genes associated with overall survival tim**e**.

Gene	G1	G2	Beta coef	HR	p-value	CI_low	CI_high	Concor-dance	Log-rank p-value
EAF2	144	143	−0.996	0.37	1.24E-08	0.26	0.52	0.62	8.83E-09
TRIM22	144	143	−0.983	0.37	5.64E-08	0.26	0.53	0.61	3.12E-08
TRBV30	144	143	−0.978	0.38	3.34E-08	0.27	0.53	0.62	1.72E-08
GPR171	144	143	−0.970	0.38	3.61E-08	0.27	0.54	0.62	2.03E-08
SAMD3	144	143	−0.942	0.39	9.47E-08	0.28	0.55	0.61	5.69E-08
OCA2	144	143	0.830	2.29	2.00E-06	1.63	3.23	0.60	1.72E-06
GSTP1	144	143	0.806	2.24	3.66E-06	1.59	3.15	0.60	3.63E-06
CS	144	143	0.760	2.14	1.79E-05	1.51	3.03	0.60	1.04E-05
PMPCA	144	143	0.750	2.12	1.44E-05	1.51	2.97	0.59	1.28E-05
FOXM1	144	143	0.702	2.02	5.84E-05	1.43	2.84	0.59	4.78E-05

Subsequently, LASSO Cox regression analysis was performed to identify the most predictive genes for survival outcomes. Using cross-validation, the optimal lambda value was determined to be 0.03, which minimized the cross-validation error. This analysis retained 17 genes (ATP11A, B2M, BISPR, CIB2, CYTL2, GBP1P1, GBP2, GCA, HEXD, HLA.DQB1, KLRC1, LRRK2.DT, MCOLN2, SLC2A5, TTYH2, WIPF1, XCL2) with non-zero coefficients, indicating their significant contribution to OS prediction. Among these, four genes were identified as risk factors, and 13 genes were identified as beneficial factors shown in Table 3. Additionally we identified 17 drugs targeting five of the selected genes, most of which act as inhibitors. Detailed information is provided in Supplementary Table S9.

**TABLE 3:** This table contains 17 prognosis-related genes in skin cutaneous melanom**a**.

Gene	Coef
ATP11A	0.035
B2M	−0.037
BISPR	−0.028
CIB2	0.005
CYTL1	0.082
GBP1P1	−0.020
GBP2	−0.015
GCA	−0.016
HEXD	−0.042
HLA.DQB1	−0.033
KLRC1	−0.028
LRRK2.DT	−0.036
MCOLN2	−0.018
SLC2A5	−0.001
TTYH2	0.049
WIPF1	−0.020
XCL2	−0.005

#### Machine learning models

To mine important genomic and epigenomic features, we used well-established feature selection methods like SVC-L1 (Guyon et al., 2002), SelecKBest (Pedregosa et al., 2011), RFE (Panda and Priyadarshi, 2022) and SFS (Esener et al., 2015). Subsequently, prediction models have been developed implementing several machine learning techniques like ExtraTrees (Geurts et al., 2006; Geeitha et al., 2024).

Various feature selection methods, including RFE, SFS, and SelectKBest with sets of 10, 20, 50, and 100 genes, were applied to identify different biomarker sets. Each set was evaluated for predictive performance, and the SVC-L1-selected features consistently showed the best performance. This set was therefore used for downstream analyses as detailed in the Supplementary Tables S2–8. The selected biomarkers were then used to train and evaluate multiple machine-learning models. Performance was assessed using metrics such as accuracy, area under the curve (AUC), specificity, sensitivity, and Matthew’s correlation coefficient (MCC). Among the models, the CatBoost algorithm emerged as the best-performing approach. It achieved an AUC of 0.90 and an MCC of 0.58 on 20 features selected by SVC-L1 based on feature importance, as shown in Table 4.

**TABLE 4:** Performance measures of the 20 mRNA genes (Primary set of biomarkers) selected through SVC-L1 for classification of survival categories in train and test datasets.

For the primary set of biomarkers												
	Train						Test					
Models	Acc	AUC	Sens	Spec	Kappa	MCC	Acc	AUC	Sens	Spec	Kappa	MCC
RF	0.67	0.88	0.67	0.67	0.57	0.56	0.66	0.86	0.66	0.65	0.55	0.55
SVM	0.66	0.86	0.66	0.67	0.56	0.55	0.65	0.85	0.65	0.64	0.54	0.53
ET	0.73	0.91	0.73	0.73	0.65	0.64	0.7	0.87	0.7	0.69	0.61	0.61
XGB	0.66	0.86	0.66	0.67	0.55	0.55	0.66	0.84	0.66	0.65	0.55	0.55
KNN	0.6	0.81	0.6	0.61	0.48	0.47	0.57	0.79	0.57	0.58	0.44	0.42
LightGBM	0.68	0.88	0.68	0.68	0.58	0.58	0.67	0.86	0.67	0.66	0.56	0.56
GB	0.58	0.78	0.58	0.58	0.44	0.44	0.58	0.78	0.58	0.57	0.44	0.44
Adaboost	0.59	0.79	0.59	0.61	0.45	0.45	0.66	0.79	0.66	0.67	0.55	0.55
Catboost	0.65	0.89	0.65	0.65	0.54	0.53	0.68	0.90	0.68	0.68	0.58	0.58

For the secondary biomarker set, the CatBoost model achieved an AUC of 0.89, as detailed in Table 5. Similarly, for the third biomarker set, CatBoost demonstrated an AUC of 0.87. Consistent performance was observed across the fourth (AUC: 0.85), sixth (AUC: 0.84), and seventh (AUC: 0.89) biomarker sets. Notably, the highest performance was achieved with the fifth biomarker set, where CatBoost attained an AUC of 0.91 and an MCC of 0.64, underscoring its superior predictive capability with this selection of biomarkers.

**TABLE 5:** Performance measures for a distinct set of biomarkers (from secondary to seventh set) selected through SVC- L1.

For the Secondary sets of biomarkers													
	Train						Test						
Models	Acc	AUC	Sens	Spec	Kappa	MCC	Acc	AUC	Sens	Spec	Kappa	MCC	
RF	0.67	0.87	0.67	0.67	0.56	0.56	0.61	0.86	0.61	0.61	0.49	0.48	
SVM	0.70	0.87	0.70	0.69	0.60	0.60	0.61	0.85	0.61	0.59	0.49	0.48	
ET	0.75	0.91	0.75	0.76	0.67	0.67	0.67	0.89	0.67	0.66	0.56	0.56	
XGB	0.66	0.85	0.66	0.66	0.55	0.54	0.61	0.84	0.61	0.60	0.49	0.48	
KNN	0.59	0.82	0.59	0.60	0.47	0.46	0.51	0.78	0.51	0.49	0.36	0.35	
LightGBM	0.69	0.88	0.69	0.69	0.59	0.58	0.66	0.85	0.66	0.65	0.55	0.55	
GB	0.57	0.78	0.57	0.57	0.42	0.42	0.57	0.79	0.57	0.56	0.43	0.42	
MLP	0.69	0.86	0.69	0.68	0.59	0.58	0.64	0.84	0.64	0.62	0.52	0.52	
AdaBoost	0.59	0.79	0.59	0.61	0.46	0.45	0.60	0.76	0.60	0.60	0.47	0.47	
CatBoost	0.66	0.89	0.66	0.65	0.56	0.55	0.68	0.89	0.68	0.66	0.58	0.58	
For the third set of Biomarkers													
	Train						Test						
Models	Acc	AUC	Sens	Spec	Kappa	MCC	Acc	AUC	Sens	Spec	Kappa	MCC	
RF	0.66	0.87	0.66	0.67	0.56	0.55	0.64	0.84	0.64	0.64	0.52	0.52	
SVM	0.65	0.85	0.65	0.65	0.54	0.53	0.63	0.84	0.63	0.61	0.51	0.50	
ET	0.72	0.91	0.72	0.73	0.62	0.62	0.66	0.87	0.66	0.64	0.55	0.55	
XGB	0.61	0.84	0.61	0.60	0.48	0.48	0.61	0.82	0.61	0.62	0.50	0.48	
KNN	0.57	0.81	0.57	0.6	0.44	0.42	0.48	0.73	0.48	0.48	0.33	0.30	
LightGBM	0.63	0.86	0.63	0.63	0.51	0.51	0.64	0.85	0.64	0.63	0.52	0.52	
GB	0.52	0.75	0.52	0.53	0.37	0.36	0.6	0.77	0.6	0.60	0.47	0.47	
MLP	0.67	0.83	0.67	0.67	0.57	0.56	0.63	0.8	0.63	0.60	0.51	0.50	
AdaBoost	0.54	0.78	0.54	0.58	0.40	0.39	0.58	0.77	0.58	0.63	0.46	0.44	
CatBoost	0.66	0.89	0.66	0.67	0.56	0.55	0.66	0.87	0.66	0.68	0.56	0.55	
For the fourth set of Biomarkers													
	Train						Test						
Models	Acc	AUC	Sens	Spec	Kappa	MCC	Acc	AUC	Sens	Spec	Kappa	MCC	
RF	0.66	0.86	0.66	0.66	0.55	0.55	0.53	0.82	0.53	0.53	0.38	0.38	
SVM	0.66	0.84	0.66	0.66	0.55	0.54	0.60	0.79	0.60	0.60	0.47	0.47	
ET	0.68	0.89	0.68	0.69	0.58	0.58	0.58	0.84	0.58	0.57	0.44	0.44	
XGB	0.61	0.82	0.61	0.61	0.48	0.48	0.57	0.82	0.57	0.57	0.43	0.42	
KNN	0.50	0.78	0.50	0.55	0.35	0.33	0.49	0.75	0.49	0.46	0.33	0.32	
LightGBM	0.61	0.84	0.61	0.61	0.49	0.48	0.56	0.81	0.56	0.55	0.41	0.41	
GB	0.54	0.77	0.54	0.54	0.39	0.39	0.52	0.76	0.52	0.51	0.37	0.36	
MLP	0.69	0.85	0.69	0.69	0.59	0.58	0.63	0.79	0.63	0.62	0.50	0.50	
AdaBoost	0.54	0.75	0.54	0.54	0.39	0.38	0.42	0.71	0.42	0.41	0.23	0.23	
CatBoost	0.67	0.88	0.67	0.67	0.56	0.56	0.60	0.85	0.60	0.61	0.48	0.47	
For the fifth set of Biomarkers													
	Train						Test						
Models	Acc	AUC	Sens	Spec	Kappa	MCC	Acc	AUC	Sens	Spec	Kappa	MCC	
RF	0.65	0.85	0.65	0.64	0.53	0.53	0.73	0.87	0.73	0.72	0.64	0.64	
SVM	0.61	0.83	0.61	0.60	0.48	0.48	0.61	0.82	0.61	0.59	0.49	0.48	
ET	0.71	0.90	0.71	0.72	0.62	0.62	0.69	0.89	0.69	0.68	0.59	0.59	
XGB	0.61	0.83	0.61	0.60	0.48	0.48	0.61	0.85	0.61	0.62	0.49	0.48	
KNN	0.56	0.78	0.56	0.56	0.42	0.42	0.51	0.75	0.51	0.50	0.36	0.35	
LightGBM	0.64	0.85	0.64	0.64	0.53	0.52	0.64	0.86	0.64	0.63	0.52	0.52	
GB	0.56	0.77	0.56	0.56	0.41	0.41	0.58	0.81	0.58	0.58	0.44	0.44	
MLP	0.67	0.84	0.67	0.66	0.56	0.56	0.61	0.77	0.61	0.62	0.49	0.48	
AdaBoost	0.66	0.89	0.66	0.64	0.55	0.54	0.73	0.91	0.73	0.72	0.64	0.64	
CatBoost	0.65	0.85	0.65	0.64	0.53	0.53	0.73	0.87	0.73	0.72	0.64	0.64	
For the sixth set of Biomarkers													
	Train						Test						
Models	Acc	AUC	Sens	Spec	Kappa	MCC	Acc	AUC	Sens	Spec	Kappa	MCC	
RF	0.65	0.86	0.65	0.67	0.54	0.54	0.56	0.81	0.56	0.58	0.41	0.41	
SVM	0.58	0.77	0.58	0.59	0.45	0.44	0.49	0.72	0.49	0.50	0.32	0.32	
ET	0.69	0.89	0.69	0.70	0.59	0.59	0.65	0.84	0.65	0.66	0.53	0.53	
XGB	0.60	0.83	0.60	0.60	0.47	0.47	0.55	0.79	0.55	0.57	0.40	0.39	
KNN	0.63	0.85	0.63	0.65	0.52	0.51	0.60	0.81	0.60	0.64	0.47	0.47	
LightGBM	0.56	0.80	0.56	0.55	0.41	0.41	0.61	0.79	0.61	0.61	0.49	0.48	
GB	0.66	0.84	0.66	0.66	0.55	0.54	0.67	0.80	0.67	0.67	0.56	0.56	
MLP	0.63	0.86	0.63	0.63	0.51	0.51	0.55	0.82	0.55	0.54	0.40	0.39	
AdaBoost	0.65	0.86	0.65	0.67	0.54	0.54	0.56	0.81	0.56	0.58	0.41	0.41	
CatBoost	0.65	0.86	0.65	0.67	0.54	0.54	0.56	0.81	0.56	0.58	0.41	0.41	
For the seventh set of Biomarkers													
	Train						Test						
Models	Acc	AUC	Sens	Spec	Kappa	MCC	Acc	AUC	Sens	Spec	Kappa	MCC	
RF	0.64	0.87	0.64	0.64	0.52	0.52	0.63	0.86	0.63	0.62	0.50	0.50	
SVM	0.61	0.80	0.61	0.63	0.48	0.47	0.61	0.74	0.61	0.61	0.49	0.48	
ET	0.70	0.89	0.70	0.70	0.60	0.59	0.65	0.87	0.65	0.64	0.53	0.53	
XGB	0.63	0.85	0.63	0.63	0.50	0.50	0.55	0.83	0.55	0.53	0.40	0.39	
KNN	0.56	0.80	0.56	0.56	0.43	0.42	0.47	0.75	0.47	0.45	0.30	0.29	
LightGBM	0.66	0.86	0.66	0.66	0.54	0.54	0.59	0.83	0.59	0.59	0.46	0.45	
GB	0.57	0.79	0.57	0.57	0.43	0.43	0.60	0.81	0.60	0.62	0.47	0.47	
MLP	0.69	0.86	0.69	0.70	0.60	0.59	0.61	0.80	0.61	0.60	0.49	0.48	
AdaBoost	0.65	0.89	0.65	0.64	0.54	0.53	0.64	0.89	0.64	0.63	0.52	0.52	
CatBoost	0.56	0.80	0.56	0.56	0.43	0.42	0.47	0.75	0.47	0.45	0.30	0.29	

**FIGURE 9:**
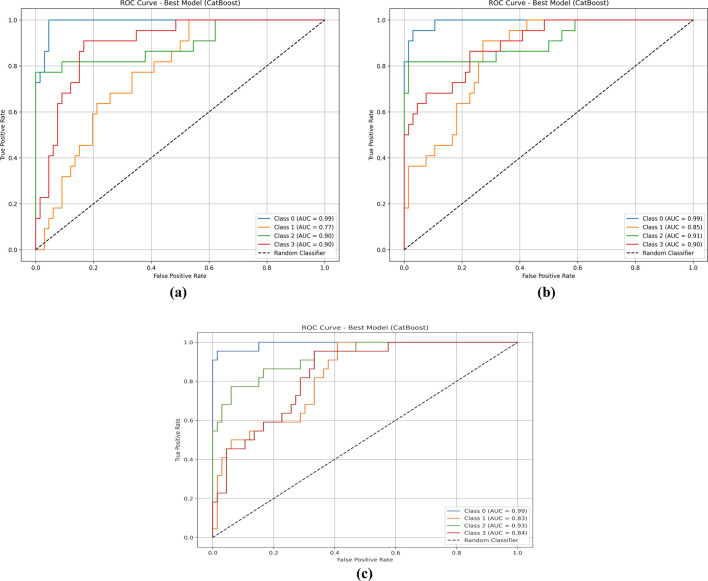
ROC curve for the best model (CatBoost) comparing true positive rate against false positive rate. Four classes are shown: **(a)** Class 0 (AUC = 0.99, blue), Class 1 (AUC = 0.77, orange), Class 2 (AUC = 0.90, green), and Class 3 (AUC = 0.90, red). **(b)** Class 0 has an AUC of 0.99, Class 1 is 0.85, Class 2 is 0.91, and Class 3 is 0.90. **(c)** Class 0 has an AUC of 0.99, Class 1 is 0.83, Class 2 is 0.93, and Class 3 is 0.84. The dashed black line represents a random classifier.

The classification performance of the CatBoost model was initially evaluated on the primary set, where it achieved outstanding results with AUC values of 0.99 for Class 0, 0.83 for Class 1, 0.93 for Class 2, and 0.84 for Class 3. Subsequently, the model was tested on the additional set for seven distinct biomarker sets, with AUC values reported for each class. For the secondary set of biomarkers, the model achieved AUC values of 0.99 for Class 0, 0.77 for Class 1, 0.90 for Class 2, and 0.90 for Class 3. In the third set of biomarkers, the AUC values were 0.98 for Class 0, 0.80 for Class 1, 0.93 for Class 2, and 0.73 for Class 3. The fourth set of biomarkers recorded AUC values of 0.80, 0.85, 0.90, and 0.87 for Classes 0, 1, 2, and 3, respectively. Similarly, the fifth set of biomarkers showed strong performance with AUC values of 0.99 for Class 0, 0.85 for Class 1, 0.91 for Class 2, and 0.90 for Class 3. The sixth set of biomarkers achieved AUC values of 0.92 for Class 0, 0.75 for Class 1, 0.90 for Class 2, and 0.77 for Class 3. Finally, the seventh set of biomarkers demonstrated AUC values of 0.95 for Class 0, 0.82 for Class 1, 0.93 for Class 2, and 0.85 for Class 3. These results, shown in Table 6, and the corresponding AUC curve presented in Figure 9, highlight the robust performance of the CatBoost model across multiple biomarkers sets, with variations in AUC values reflecting differences in the predictive potential of the biomarker sets.

**TABLE 6:** AUC for each class using the best-performing model on all seven sets of biomarkers.

Class	Primary	Secondary	Third	Fourth	Fifth	Sixth	Seventh
0	0.99	0.99	0.98	0.90	0.99	0.92	0.95
1	0.83	0.77	0.80	0.80	0.85	0.75	0.82
2	0.93	0.90	0.93	0.88	0.91	0.90	0.93
3	0.84	0.90	0.73	0.82	0.90	0.77	0.85

The AUC values in Table 6 illustrate the predictive performance of the best-performing model across seven biomarker sets for survival classes (0–1 year, 1–3 years, 3–5 years, and >5 years). For Class 0 (0–1 year survival), the AUC values are consistently high, ranging from 0.90 to 0.99, with the primary, secondary, and fifth biomarker sets achieving the highest AUC (0.99). This indicates exceptional accuracy in identifying patients with the shortest survival times, highlighting these biomarker sets as highly effective for predicting high-risk patients who may require immediate clinical intervention. For Class 1 (one to three years survival), the AUC values are moderate, ranging from 0.75 to 0.85, with the fifth biomarker set demonstrating the highest predictive accuracy (0.85) and the sixth set performing the least effectively (0.75). These results suggest moderate success in predicting mid-range survival groups, indicating room for improvement in this category.

For Class 2 (three to five years survival), the AUC values are consistently high across all biomarkers sets, ranging from 0.88 to 0.93, with the primary, third, and seventh sets achieving the highest AUC (0.93). This consistency demonstrates the robustness of these sets in predicting intermediate survival times, making them promise for accurate prognostic stratification in this group. For Class 3 (>5 years survival), the AUC values show more variation, ranging from 0.73 to 0.90. The secondary and fifth biomarker sets achieve the highest performance (0.90), while the third set shows the lowest (0.73), indicating that while some sets are effective for long-term survival prediction, others may lack the necessary features to differentiate this class accurately.

Overall, the primary biomarker set stands out for its exceptional performance across all classes, particularly for Class 0 and Class 2 (AUC = 0.99 and 0.93, respectively), making it the most reliable set for survival prediction. The fifth biomarker set demonstrates high performance, particularly for Classes 0 and 3 (AUC = 0.99 and 0.90), showing its potential for identifying high-risk and long-term survivors. The results reveal that the highest predictive accuracy is observed for Class 0, suggesting the model excels in identifying high-risk patients. Moderate performance for Class 1 indicates challenges in predicting mid-range survival groups, while high accuracy for Class 2 and variable performance for Class 3 highlights the model’s strengths. These findings emphasize the potential of these biomarker sets in predicting survival outcomes in SKCM patients, with the primary and fifth sets showing the best performance.

### Ensemble models

The Voting and Stacking classifiers were applied to the 20 prognostic genes from the primary set. The Voting classifier combined RF, ET, and LightGBM, achieved an AUC of 0.87 and an MCC of 0.56. The Stacking classifier was evaluated using Logistic Regression as the meta-model with RF and ET as base models and CatBoost as the meta-model with RF, ET, and XGB as base models. These stacking models achieved AUC values of 0.88, with MCC scores of 0.61. The results are summarized in Table 7.

**TABLE 7:** Performance on 20 genes selected using SVC-L1.

Combination of RF, ET and LightGBM												
	Train						Test					
Classifier	Acc	AUC	Sens	Spec	Kappa	MCC	Acc	AUC	Sens	Spec	Kappa	MCC
Voting	0.69	0.90	0.69	0.70	0.59	0.59	0.67	0.87	0.67	0.66	0.56	0.56
LR as meta-model and RF, ET as base-models												
Stacking	0.74	0.91	0.74	0.74	0.66	0.65	0.70	0.88	0.70	0.70	0.61	0.61
Catboost as meta-model and RF, ET, and XGB as base-models												
Stacking	0.69	0.89	0.69	0.71	0.59	0.59	0.64	0.88	0.64	0.64	0.52	0.52

The best existing method, which focused on IAGS, achieved an AUC of 0.88. In our study, four out of seven biomarker sets performed even better, with AUC values higher than 0.88. This demonstrates that our approach is more effective in identifying prognostic biomarkers with higher accuracy.

### Validation on a new dataset

The best-performing model in our study, CatBoost was used to evaluate the prognostic potential of the selected biomarkers. Using the primary biomarker set, which included 15 matched genes, the model achieved an AUC of 0.85 on the training set and 0.83 on the test set, indicating strong predictive performance. Class-wise AUCs for this model were: Class 0 (AUC = 0.76), Class 1 (AUC = 0.77), Class 2 (AUC = 0.93), and Class 3 (AUC = 0.85). Similarly, using the third biomarker set with 12 overlapping genes, the model achieved an AUC of 0.85 on training and 0.86 on testing. Class-wise AUCs for this set were: Class 0 (AUC = 0.79), Class 1 (AUC = 0.78), Class 2 (AUC = 0.90), and Class 3 (AUC = 0.97), further highlighting the robustness and reliability of the selected biomarkers. To provide biological context for our biomarker gene sets, we conducted Reactome pathway enrichment analysis, identifying significantly enriched pathways—including ȁCRNA Polymerase I Promoter Opening,” ȁPackaging of Telomere Ends,” ȁPost-translational Protein Modification,” and ȁMetabolism of Proteins” which are biologically relevant to melanoma, with detailed results for all seven biomarker sets presented in Supplementary Figures S1-S7, where bubble color denotes adjusted p-values and bubble size indicates the number of enriched genes.

## Discussion

Melanoma is the most lethal and deadliest form of skin cancer (Lombard et al., 2019). The prognosis is often poor due to early metastasis, which remains the primary cause of death in affected individuals (Sun et al., 2019). Therefore, early detection of SKCM and effective stratification of risk assessment are crucial for timely treatment and the improvement of survival rates. As one of the most immunogenic tumors, the role of immune regulation and the potential of immunotherapy in SKCM have consistently been central to research and clinical discussions (Marzagalli et al., 2019). We utilized the TCGA dataset to identify potential prognostic biomarkers for SKCM. Through *in silico* analyses of gene expression profiles from TCGA, we aimed to uncover key genes associated with patient survival, disease progression, and therapeutic response, ultimately enhancing the prognostic accuracy for SKCM patients.

Our study takes a comprehensive approach, focusing on a broad range of genes rather than targeting specific types or pathways. This strategy aims to identify distinct sets of biomarkers associated with overall survival in SKCM. All available genes were analyzed using statistical and machine-learning methods to uncover meaningful prognostic biomarkers. Based on the median OS time, a cohort of 287 patients with available survival data was divided into high- and low-risk groups. In our analysis, genes correlated with OS time were first identified. Pearson correlation analysis revealed several genes with significant correlations to OS, including CREG1 and PCGF5, which showed positive correlations, and AC008687.4 and TTYH2, which demonstrated negative correlations. Next, the top 50 positively correlated genes were selected for further exploration. These genes were enriched in various biological processes and pathways, including lipid translocation and phospholipid translocation. Reactome pathway analysis further revealed their involvement in key pathways such as *'*Ion Transport By P-type ATPases’ and 'Ion Channel Transport.'

Our univariate Cox regression analysis revealed a substantial number of genes significantly associated with OS time, with a total of 4324 genes found to have p-values below 0.01. Among these, 1,264 genes were identified with a hazard ratio (HR > 1), indicating potential risk factors, while 3,060 genes were associated with HR < 1, suggesting a protective effect on survival. These results emphasize the complexity of SKCM’s molecular landscape, where multiple genes may promote or inhibit tumor progression and patient survival. The gene list was further refined to 17 genes with non-zero coefficients by applying LASSO Cox regression. The identification of risk and beneficial factors among the 17 selected genes is another key finding of our study. Four of these genes were classified as risk factors, while the remaining 13 genes were classified as beneficial factors. This classification not only underscores the diverse nature of the molecular drivers of SKCM but also offers potential biomarkers for therapeutic targeting. For instance, CYTL1 expression was progressively upregulated in normal skin, nevi or malignant nevi, and melanoma (Tao et al., 2023). Similarly, B2M (Beta-2-Microglobulin) is associated with worse OS and disease-specific survival (DSS) in SKCM, acting as a hazardous factor by contributing to tumor progression and immune evasion (Zhang H. et al., 2021). GBP2 was downregulated in SKCM and Low GBP2 expression was positively correlated with poor prognosis of SKCM (Ji et al., 2021). The Expressions of HLA Class II (HLA-DQB1) genes were Upregulated in Cutaneous Melanoma (Chen et al., 2019). KLRC1 expression in SKCM is associated with immune cell infiltration, highlighting its role in immune surveillance and tumor microenvironment modulation (Kamiya et al., 2019). MCOLN2 (TRPML2) has not been extensively studied in SKCM. However, its upregulation in other cancers like prostate tumors and its association with poor prognosis suggests it might play a role in melanoma progression by potentially influencing tumor growth, invasion, or immune evasion (Li and Zhu, 2023) in three distinct malignancies that contained SKCM, SARC, and THCA, SLC2A5 acted as a protective factor (Liu et al., 2024). WIPF1 upregulated in early melanoma (Zia et al., 2023). XCL2 shows high expression in SKCM (Xiong et al., 2020). The DGIdb database to predict drug targets and found 17 drugs acting on five key genes linked to survival outcomes. While these findings suggest potential therapeutic targets, clinical validation is needed to confirm their relevance. This exploratory analysis provides a basis for future research on the clinical applicability of the identified gene signature.The top 20 mRNA genes selected using SVC-L1 achieved an AUC of 0.91, indicating that machine-learning techniques can be effectively utilized for survival prediction in SKCM. These results are consistent with other machine learning-based studies in melanoma, which have demonstrated the utility of advanced algorithms, such as support vector machines and random forests, for identifying prognostic biomarkers (Bhalla et al., 2019). So, the primary set of biomarkers associated with skin cutaneous melanoma includes TEKT5, ZNF154, H2AC14, BX284668.6, MYCNOS, STUM, SERTM2, RPSAP18, REG4, PSCA, PAEP, ACTR3C, MSLN, MRPS18AP1, ISLR, IL37, IGLV3.16, H2BC11, GPR25, and MTND4P35. PSCA (Prostate Stem Cell Antigen) is upregulated in SKCM, as suggested by its positive correlation with genes related to RNA modifications, indicating an active role in the tumor’s biological processes (Wang et al., 2024). PAEP (Progestogen-Associated Endometrial Protein) is upregulated and correlates with poor survival outcomes in SKCM (Yan et al., 2020). ACTR3C mutations, such as p. Gly58Arg or p.Gly58Glu, might alter protein function and affect key cellular processes such as cell signaling, motility, or adhesion, which are crucial in melanoma progression (Srinivasan et al., 2021). MSLN expression is downregulated in SKCM (Li et al., 2022). ISLR is also downregulated and negatively correlated with Tumor Mutation load (TMB) (Zhang C. et al., 2021). STUM is a biomarker for response to treatment with Nivolumab (immune checkpoint inhibitors) (Bannon et al., 2024). SERTM2 gene alterations are frequently observed in melanoma, suggesting a potential role in tumor progression and immune evasion. These alterations may impact pathways related to melanoma growth, metastasis, and immune response modulation (Niu et al., 2024). Secondary biomarkers play a vital role in other cancers but are not directly related to SKCM (He et al., 2020; Jia et al., 2021; Larsson et al., 2022; Severgnini et al., 2022; Bisaccia et al., 2023). For the fifth set of biomarkers, we did not find any direct evidence of their specific role in SKCM. These genes are newly identified in our study and may serve as potential prognostic biomarkers (Yang W. et al., 2021; Ding et al., 2022; Fang et al., 2022). For Class 0 (0–1 year survival), the AUC values were exceptionally high, ranging from 0.90 to 0.99, with the primary, secondary, and fifth biomarker sets achieving the highest AUC of 0.99. This suggests that these sets are highly effective in identifying high-risk patients with the shortest survival times, making them valuable for early-stage risk assessment and timely clinical intervention. In contrast, for Class 1 (one to three years survival), the AUC values ranged from 0.75 to 0.85, with the fifth biomarker set showing the best performance (AUC = 0.85). The moderate AUC for this class indicates that predicting mid-range survival times remains challenging, and further refinement of the biomarkers may be needed to improve accuracy in this group.

For Class 2 (three to five years survival), the AUC values were consistently high across all sets, ranging from 0.88 to 0.93, with the primary, third, and seventh sets achieving the highest AUC of 0.93. This highlights the robustness of these biomarker sets in predicting intermediate survival, making them useful for more accurate prognostic stratification. However, for Class 3 (>5 years survival), the AUC values showed more variation, ranging from 0.73 to 0.90, with the secondary and fifth sets performing the best (AUC = 0.90). The lower performance for some sets in this class suggests that further refinement is needed to capture the specific molecular features associated with long-term survival.Overall, the primary and fifth biomarker sets showed better performance compared to the others and were particularly effective in predicting high-risk patients. The robustness of the selected biomarkers was further validated using the GSE65904 dataset, where CatBoost consistently demonstrated strong predictive performance on both the 15-gene and 12-gene sets. Notably, all survival classes showed significant AUCs, with Class 2 and Class 3 achieving particularly high values, highlighting the model’s ability to predict long-term survival outcomes. CatBoost’s superior performance is likely due to its capability to handle high-dimensional gene expression data, capture complex nonlinear interactions between genes, and reduce overfitting, making it a robust tool for prognostic modeling in cancer.

A key limitation of this study is its reliance on computational analysis of publicly available datasets, without experimental validation. Although the results are promising, they should be viewed as preliminary. Confirming the biological relevance of the identified biomarkers will require further validation in wet-lab settings, such as gene expression profiling in independent patient samples.

## Conclusion

This study identified seven distinct biomarker sets and developed a robust prognostic model for predicting overall survival in skin cutaneous melanoma (SKCM). Using machine learning techniques, the most relevant biomarkers associated with patient prognosis were selected. The model effectively classified patients into different survival categories based on OS time, supporting its potential use in clinical prognosis prediction. These findings demonstrate the value of multiple biomarker sets in understanding the prognostic landscape of SKCM. However, further validation and clinical studies are required to confirm their applicability in real-world settings.

## Data Availability

Publicly available datasets were analyzed in this study. This data can be found here: https://xenabrowser.net/datapages/?cohort=GDC%20TCGA%20Melanoma%20(SKCM)&removeHub=https%3A%2F%2Fxena.treehouse.gi.ucsc.edu%3A443 (TCGA-SKCM).

## Abbreviations

SKCM, Skin Cutaneous Melanoma; OS, Overall survival; IAGS, Invasion-associated genes; TCGA The Cancer Genome Atlas; FRG,Ferroptosis-related gene; DGIdb, Drug Gene Interaction Database; ML, Machine Learning; AUROC, Area Under the Receiver Operating Characteristic.
