# Supplementary material for: Identification of multiple prognostic biomarker sets for risk stratification in SKCM
Source: Front Bioinform. 2026 Jan 7;5:1624329. doi: 10.3389/fbinf.2025.1624329 (PMC12819672; doi:10.3389/fbinf.2025.1624329)
Supplement: Supplementary file 2 [file DataSheet1.docx]

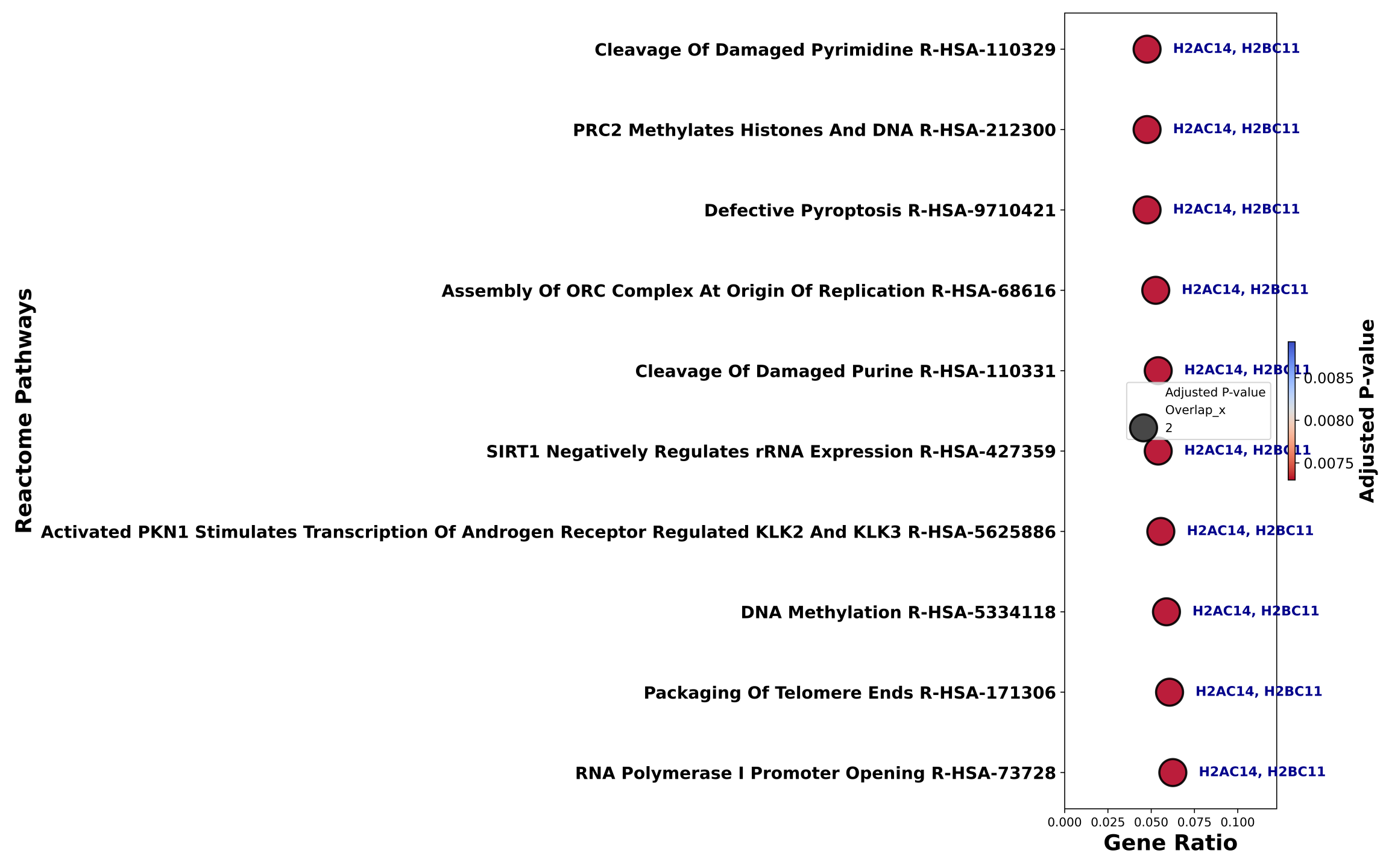


**Figure S1.** Reactome pathway enrichment analysis of the primary biomarker set. The top significantly enriched pathways are shown, ranked by –log10(p-value).


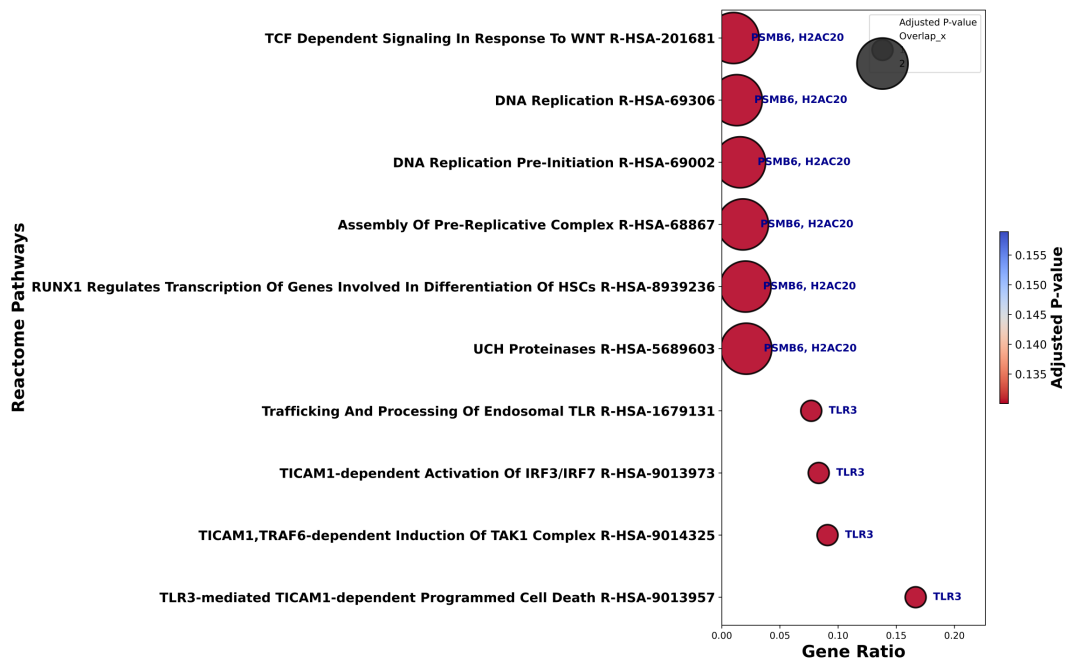


Figure S2. Reactome pathway enrichment analysis of the second biomarker set. The top significantly enriched pathways are shown, ranked by –log10(p-value).


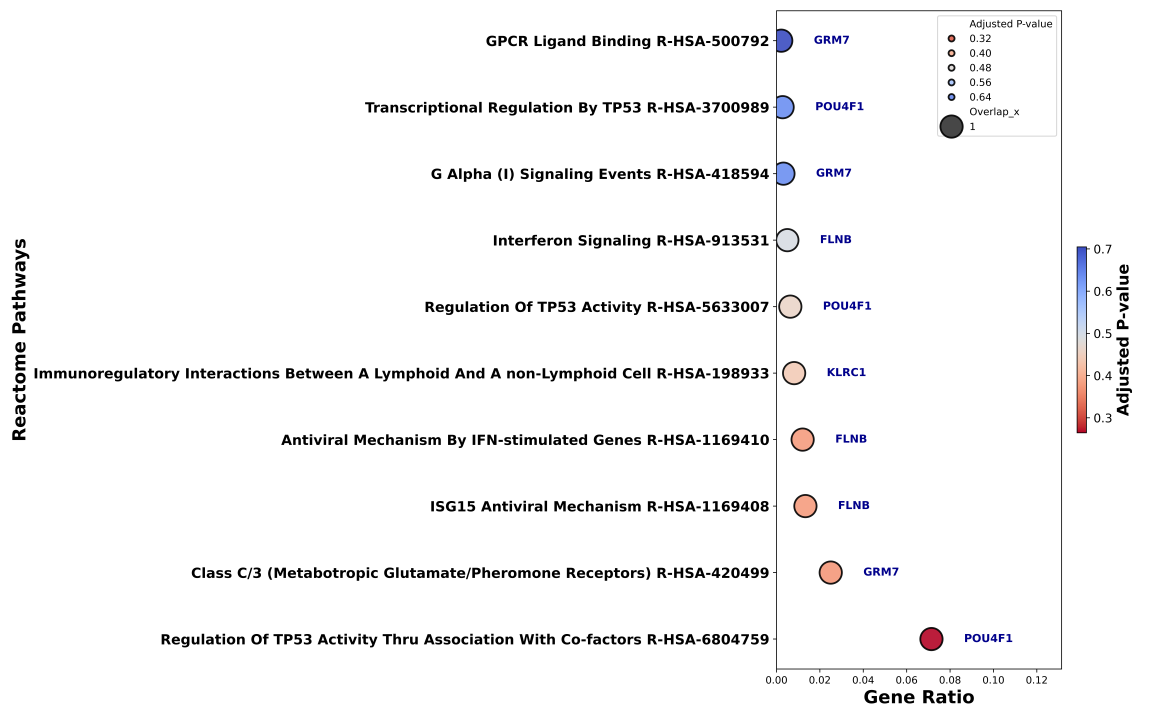


**Figure S3.** Reactome pathway enrichment analysis of the third biomarker set. The top significantly enriched pathways are shown, ranked by –log10(p-value).


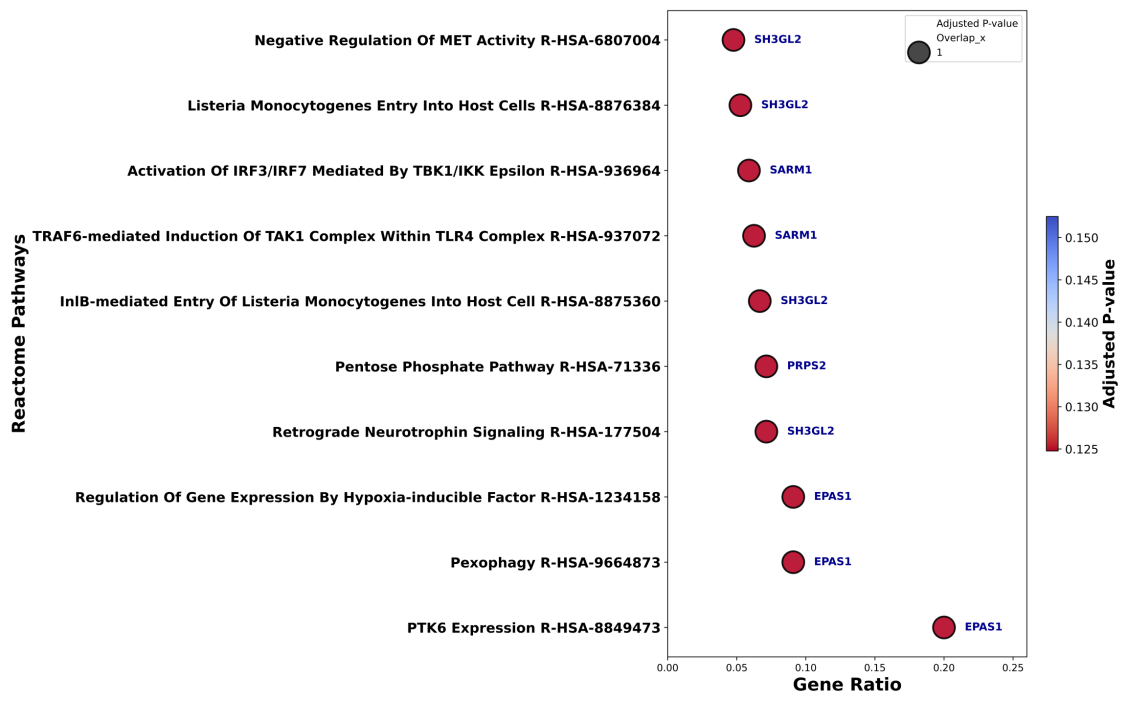


**Figure S4.** Reactome pathway enrichment analysis of the fourth biomarker set. The top significantly enriched pathways are shown, ranked by –log10(p-value).


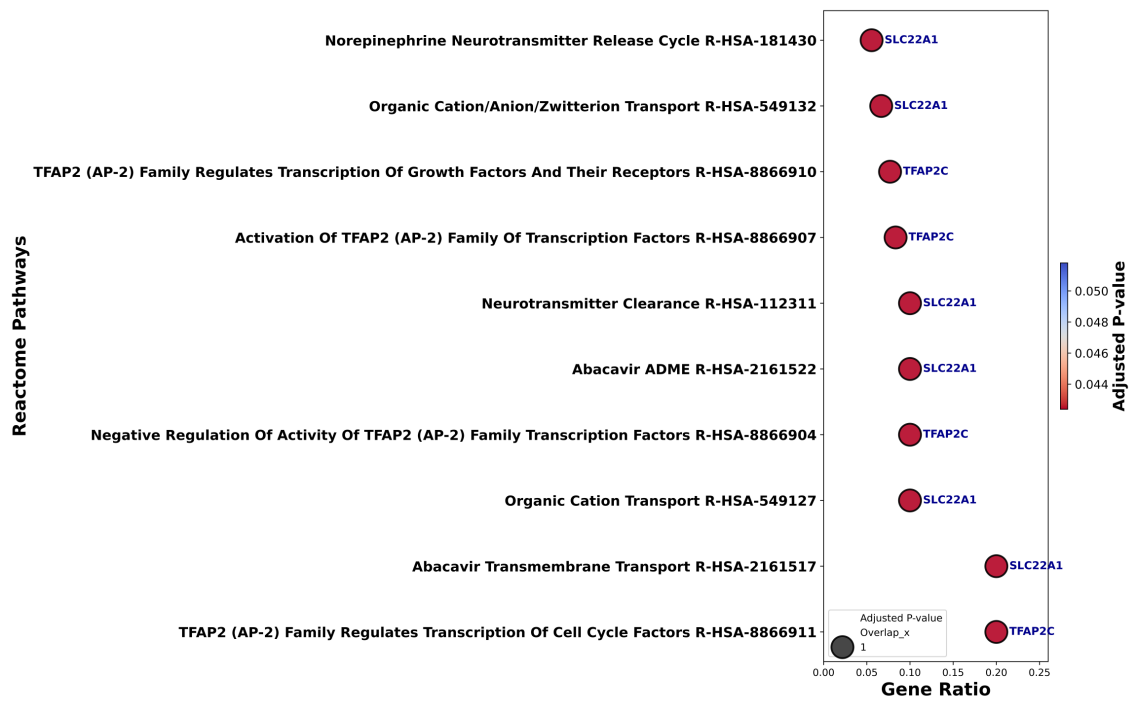


**Figure S5.** Reactome pathway enrichment analysis of the fifth biomarker set. The top significantly enriched pathways are shown, ranked by –log10(p-value).


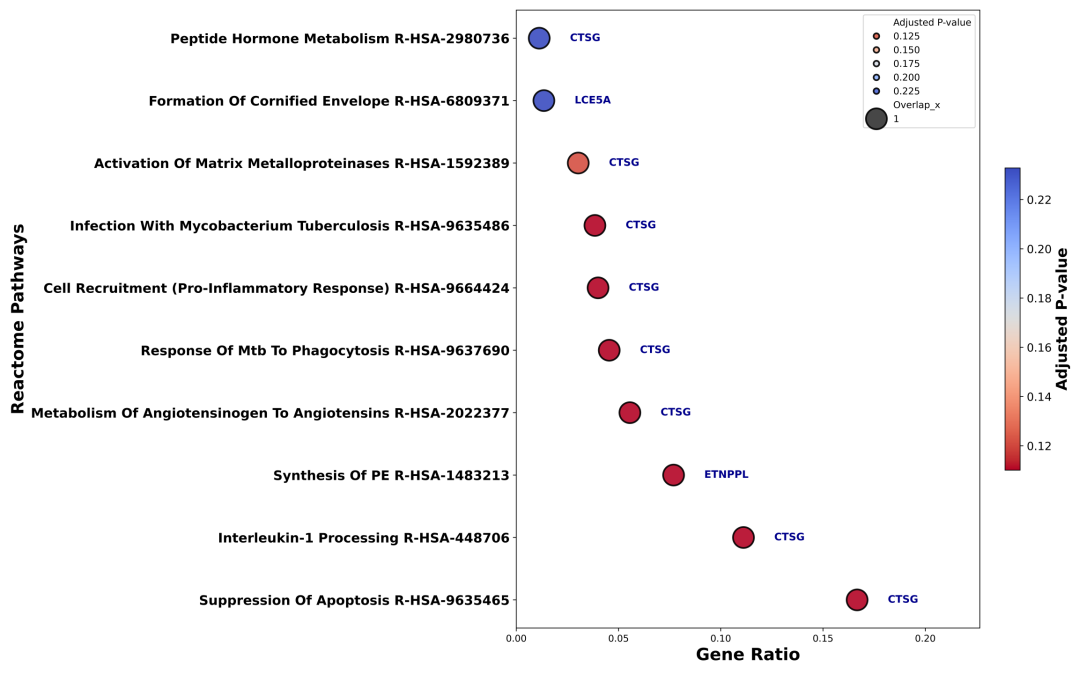


**Figure S6.** Reactome pathway enrichment analysis of the sixth biomarker set. The top significantly enriched pathways are shown, ranked by –log10(p-value).


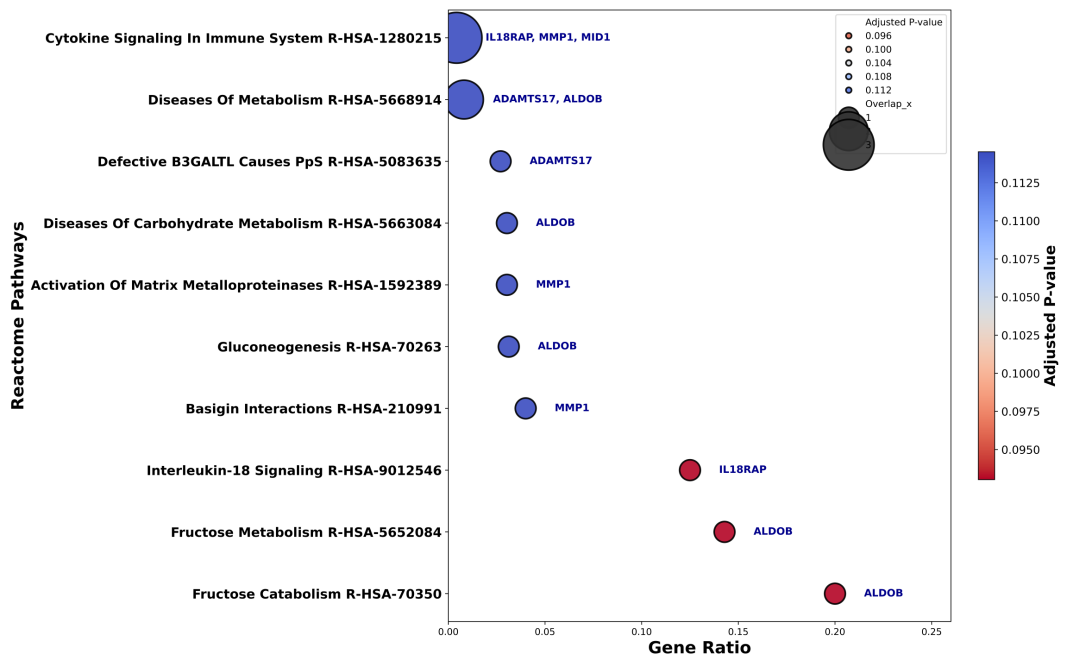


**Figure S7.** Reactome pathway enrichment analysis of the seventh biomarker set. The top significantly enriched pathways are shown, ranked by –log10(p-value).
